# New imaging findings of tumor invasion into brain tissue: “Peritumoral Hyperintense Enhancement Sign”

**DOI:** 10.3389/fnhum.2025.1592543

**Published:** 2025-05-13

**Authors:** Xinyi Mao, Jianye Li, Xuejun Zheng, Yujun Wang, Jie Gao, Chunlong Fu, Xin Li, Ming Liang, Xiangping Wang, Tiantian Qiu, Haijun Du, Chen Xue, Yufeng Liu

**Affiliations:** ^1^Department of Radiology, The First Affiliated Hospital of Zhejiang Chinese Medical University (Zhejiang Provincial Hospital of Chinese Medicine), Hangzhou, China; ^2^Zhejiang Chinese Medical University, Hangzhou, China; ^3^Department of Radiology, Gutian County Hospital, Ningde, China; ^4^Department of Radiology, Linyi People’s Hospital, Linyi, China; ^5^Department of Radiology, Sanbo Brain Hospital, Capital Medical University, Beijing, China; ^6^Department of Radiology, Dongyang Hospital of Wenzhou Medical University, Dongyang, China; ^7^Department of Radiology, Xingtai Central Hospital, Xingtai, China; ^8^Traditional Chinese Medicine Hospital of Zaozhuang, Zaozhuang, China; ^9^First People's Hospital of Linping District (Linping Campus, The Second Affiliated Hospital of Zhejiang University School of Medicine), Hangzhou, China; ^10^Department of Intervention Radiology, Dongyang Hospital of Wenzhou Medical University, Dongyang, China; ^11^Department of Radiology, The Affiliated Brain Hospital of Nanjing Medical University, Nanjing, China

**Keywords:** “Peritumoral Hyperintense Enhancement Sign”, Gd-DTPA, MRI, meningiomas, brain metastases

## Abstract

**Introduction:**

This study investigated the MRI characteristics of meningiomas and brain metastases, exploring the relationship between the “Peritumoral Hyperintense Enhancement Sign” and brain invasion, and its clinical implications for treatment planning.

**Methods:**

A multicenter retrospective analysis was conducted on 24 cases (17 brain metastases and 7 meningiomas), examining the MRI features of the “Peritumoral Hyperintense Enhancement Sign” and corresponding histopathological characteristics.

**Results:**

All cases demonstrated peritumoral enhancement: 8 cases exhibited flame-shaped enhancement, 12 showed crescentic enhancement, and 4 displayed both patterns. Histopathological analysis confirmed brain invasion in regions showing abnormal enhancement.

**Conclusion:**

The “Peritumoral Hyperintense Enhancement Sign” not only serves as a valuable indicator of brain invasion and provides guidance for clinical target delineation in treatment planning, but also facilitates more precise treatment planning and may contribute to improved prognostic assessment and reduced recurrence risk.

## 1 Introduction

Meningiomas and brain metastases represent common intracranial neoplasms that frequently exhibit brain invasion (Brokinkel et al., 2017; Achrol et al., 2019). MRI remains the preferred diagnostic modality for these tumors (Park et al., 2019; Yaltırık Bilgin et al., 2023). Through extensive image analysis, a distinct imaging feature was identified: highly enhanced areas appearing in crescentic or flame-shaped patterns within adjacent brain tissues with enhancement patterns distinct from the main tumor mass, accompanied by extensive peritumoral edema. We term this phenomenon the ““Peritumoral Hyperintense Enhancement Sign.”” In contrast to conventional radiological markers that rely primarily on tumor heterogeneity, the ““Peritumoral Hyperintense Enhancement Sign”” emphasizes the differential analysis between abnormal peritumoral signal characteristics and the tumor’s intrinsic signal pattern. This sign serves as a radiological indicator of brain invasion, likely attributable to the tumor’s aggressive biological behavior. At cellular and histological levels, tumor infiltration into adjacent brain parenchyma triggers a reactive inflammatory response, compromises blood–brain barrier integrity, and promotes vascular permeability. These pathophysiological processes manifest as extensive cerebral edema, while contrast enhancement reflects gadolinium extravasation in the affected areas. The sign’s distinctive appearance stems from the heterogeneous microenvironment where infiltrating tumor cells coexist with native brain tissue, creating signal characteristics that diverge from those of the primary tumor mass. Histopathologically, tumor cells infiltrating along white matter tracts generate flame-shaped enhancement patterns, while diffuse parenchymal invasion produces crescent-shaped or irregular enhancement configurations.

## 2 Methods and patients

### 2.1 Clinical data

A multicenter retrospective study analyzed 24 patients with intracranial tumors, comprising 17 cases of pathologically confirmed brain metastases and 7 cases of confirmed meningiomas. Among patients with brain metastases (7 males, 10 females; age range 39–76 years, mean 61.2 ± 11.4 years), clinical manifestations included headache (*n* = 9), nausea and vomiting (*n* = 3), dizziness (*n* = 4), impaired consciousness with limb convulsions (*n* = 2), speech impairment (*n* = 2), limb weakness (*n* = 5), and gait instability (*n* = 3). The meningioma group (6 males, 1 females; age range 31–73 years, mean 53.7 ± 15.3 years) presented with headache (*n* = 2), dizziness (*n* = 3), limb weakness (*n* = 6), gait instability (*n* = 1), and speech impairment (*n* = 1).

### 2.2 Method

#### 2.2.1 Imaging method

All 24 cases underwent conventional and contrast-enhanced MRI examinations using both Siemens 1.5 T (Siemens Magnetom Vision Plus 1.5 T) and 3.0 T (Siemens Verio, Erlangen, Germany) scanners. Imaging was performed using a standard 8-channel head coil, including conventional MRI sequences, DWI, and contrast-enhanced scans. The conventional MRI protocol comprised axial T1-weighted imaging (TR 500 ms, TE 7.4 ms), axial T2-weighted imaging (TR 9000 ms, TE 120 ms), and T2-FLAIR sequences, with slice gap of 1.5 mm, slice thickness of 5 mm, and field of view of 240 mm × 240 mm. DWI parameters were: b-value = 1,000 s/mm^2^, TR 4600 ms, and TE 90 ms. For contrast-enhanced imaging, Gd-DTPA was administered, followed by T1-weighted scans in axial, sagittal, and coronal planes.

#### 2.2.2 Pathological methods

All tumor specimens were prepared as pathological sections from the tumor-brain interface planes observed on MRI for correlation analysis between MRI findings and histopathological features.

#### 2.2.3 Imaging analysis

Two radiologists (one senior attending physician and one resident) independently reviewed the MRI images. Following their analysis, they reached consensus on tumor location, peritumoral edema, enhancement patterns, and final diagnosis.

#### 2.2.4 Pathological analysis

Two pathologists independently examined the pathological specimens and reached consensus on tumor classification and the presence of brain invasion before making their final diagnosis. Brain invasion was histologically defined as the presence of tumor tissue within adjacent brain tissue without an intervening layer of connective tissue.

## 3 Results

The clinical data and imaging findings of 24 patients were summarized in Table 1, with histopathological results presented in Table 2.

**Table 1:** The clinical data and imaging findings of 24 patients.

Patient	Sex/Age (y)	Clinical presentation	Site of tumor	Peritumoral edema	Enhancement of brain tissue adjacent to tumor body
1	F/42	Headache	Right temporal lobe	Positive	Positive
2	M/67	Headache	Right parieto-occipital lobe	Positive	Positive
3	M/69	Headache, Weakness of lower limbs	Right occipital lobe	Positive	Positive
4	M/73	Headache, Nausea, Vomiting	Left frontal lobe	Positive	Positive
5	M/63	Headache, Slurred speech, Weakness of lower limbs	Right frontal lobe	Positive	Positive
6	F/46	Dizziness	Right cerebellar hemisphere	Positive	Positive
7	F/70	Headache, Vomiting, Limb weakness	Right occipital lobe	Positive	Positive
8	F/54	Epilepsy	Bilateral frontal lobe	Positive	Positive
9	F/39	Dizziness, Nausea, Vomiting	Right cerebellar hemisphere	Positive	Positive
10	F/52	Epilepsy	Left parietal lobe	Positive	Positive
11	M/74	Headache, Limb weakness	Right frontal–Parietal lobe	Positive	Positive
12	F/68	Memory loss, Limb weakness	Right parietal lobe	Positive	Positive
13	F/66	Unsteady walking	Right cerebellar hemisphere	Positive	Positive
14	F/49	Dizziness, Unsteady walking	Left frontal lobe	Positive	Positive
15	M/70	Headache, Dizziness, Unsteady walking	Right centrum semiovale, Right cerebellar hemisphere	Positive	Positive
16	F/63	Hemiparesis, Slurred speech	Left frontal lobe	Positive	Positive
17	M/76	Headache, Dizziness	Right cerebellar hemisphere	Positive	Positive
18	M/69	Limb weakness	Left parietal area	Positive	Positive
19	M/73	Limb weakness	Left frontal area	Positive	Positive
20	M/31	Headache, Dizziness, Unsteady walking	Left cerebellopontine angle	Positive	Positive
21	F/49	Dizziness, Weakness of lower limbs	Right frontal area	Positive	Positive
22	M/46	Blurred vision, Weakness of lower limbs, Slurred speech	Right frontal area	Positive	Positive
23	M/69	Limb weakness	Left frontal area	Positive	Positive
24	M/39	Limb weakness	Left frontoparietal area	Positive	Positive

**Table 2:** Histopathological results of “Peritumoral Hyperintense Enhancement Sign” in all cases.

Patient	Tumor type	Brain invasion
1	Endocervical adenocarcinoma	Positive
2	Poor differentiated bladder urothelial carcinoma	Positive
3	Lung adenocarcinoma	Positive
4	Lung adenocarcinoma	Positive
5	Lung adenocarcinoma	Positive
6	Lobular carcinoma	Positive
7	Colon adenocarcinoma	Positive
8	Breast cancer	Positive
9	Breast cancer	Positive
10	Breast cancer	Positive
11	Breast cancer	Positive
12	Lung adenocarcinoma	Positive
13	Endocervical adenocarcinoma	Positive
14	Endocervical adenocarcinoma	Positive
15	Endocervical adenocarcinoma	Positive
16	Endocervical adenocarcinoma	Positive
17	Malignant Cutaneous Melanoma	Positive
18	Anaplastic meningioma	Positive
19	Atypical meningioma	Positive
20	Anaplastic meningioma	Positive
21	Atypical meningioma	Positive
22	Atypical meningioma	Positive
23	Atypical meningioma	Positive
24	Atypical meningioma	Positive

### 3.1 Imaging findings

**Figure 1:**
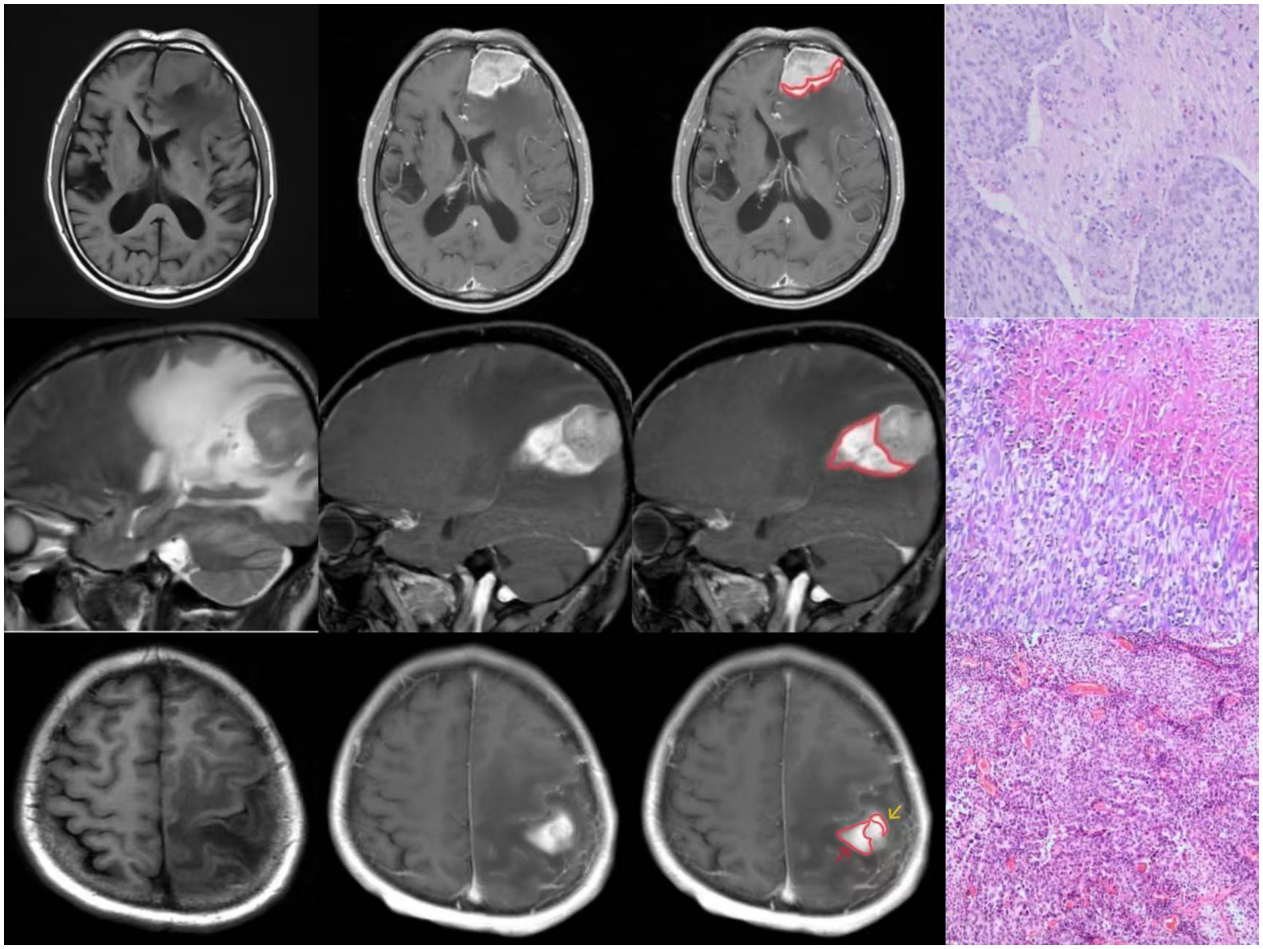
Three cases of “Peritumoral Hyperintense Enhancement Sign” with pathologically confirmed brain invasion.

All tumors demonstrated abnormal enhancement area adjacent to brain tissue, with signal characteristics distinct from the main tumor mass (Figure 1).

### 3.2 Pathological findings

Histopathological examination confirmed malignancy in all cases. Microscopic analysis revealed tumor tissue arranged in sheet-like patterns, characterized by large cells with abundant cytoplasm. The nuclei showed marked pleomorphism, varying in size with round to oval shapes, and demonstrated significant cellular atypia. Numerous mitotic figures were observed. The tumor tissue exhibited extensive areas of degeneration and necrosis, with evidence of tumor cell infiltration into the adjacent brain tissue (Figure 1).

## 4 Discussion

The “Peritumoral Hyperintense Enhancement Sign” is defined as a crescent-shaped or flame-like hyperintense signal adjacent to the tumor on contrast-enhanced scans in both meningiomas and metastases. This distinct enhancement pattern, which differs significantly from the primary tumor mass, is typically accompanied by extensive peritumoral edema. The abnormal signal demarcation exhibits a distinct boundary at the tumor interface, while demonstrating a gradual, indistinct transition toward the adjacent brain parenchyma. Histopathological analysis confirms brain invasion in these regions. The mechanism likely involves aggressive tumor invasion at cellular and histological levels, triggering reactive inflammation in brain tissue, increased vascular permeability, and blood–brain barrier disruption. This process leads to both water leakage (causing peritumoral edema) and gadolinium extravasation (producing enhancement). The unique signal characteristics arise from the coexistence of scattered tumor cells with brain tissue in the peritumoral region. This area exhibits distinct signal characteristics compared to the primary tumor mass on non-contrast imaging. Tumor infiltration along nerve fiber bundles often manifests as flame-like projections, while amorphous infiltration patterns form irregular or crescentic configurations.

Since meningioma and metastatic tumor are two distinct tumors with histopathological differences, the formation mechanism of the “Peritumoral Hyperintense Enhancement Sign” is different, and the impact on the treatment of the two tumors is also different, the next section will describe meningiomas and metastatic tumors separately.

### 4.1 Meningioma

Meningiomas, among the most prevalent adult intracranial tumors, are diagnosed based on clinical symptoms, imaging findings, and histopathology (Brokinkel et al., 2017; WHO Classification of Tumours Editorial Board, 2021). Atypical clinical presentations and the lack of mandatory histologic verification in some cases complicate the assessment of brain invasion. Histologic confirmation further depends on sampling peritumoral brain tissue, underscoring the importance of non-invasive imaging for accurate grading (Wang et al., 2024; Li et al., 2021). The fifth edition of the WHO Classification of Tumors of the Central Nervous System designates meningiomas with brain invasion as grade II (Park et al., 2019), a category associated with higher recurrence rates and poorer prognosis. Conventional MRI features—peritumoral edema, heterogeneous contrast enhancement, and irregular tumor margins—correlate with brain invasion (Jiang et al., 2023; Joo et al., 2021; Ong et al., 2021; Adeli et al., 2018). The “Peritumoral Hyperintense Enhancement Sign,” integrating these features, enhances diagnostic reliability for identifying brain invasion.

Various imaging features at the tumor-brain interface of invasive meningiomas have been described in the literature, including “finger-like protrusions,” “pseudopod sign,” and “mushroom sign.” The finger-like protrusions and pseudopod sign characterize morphological abnormalities of meningioma growth patterns. The mushroom sign, however, has been inconsistently defined across studies. Adeli et al. described it as mushroom-shaped protrusions on the tumor surface (Adeli et al., 2018), while Jiang et al. defined it as an enhancement band of spherical tumors invading peripherally along dural appendages—extending farther, appearing thicker, and longer than the typical dural tail sign—with associated roughening of the proximal brain surface (Jiang et al., 2023). The latter interpretation considers this sign a variant of the dural tail sign. Notably, neither interpretation establishes clear histopathological correlations. In contrast, the “Peritumoral Hyperintense Enhancement Sign” described in our study clearly differentiates abnormal signal areas from the tumor itself, providing more specific radiological evidence of brain invasion.

The “brain-meningioma interface” represents the critical boundary that either facilitates or prevents brain invasion in these intracranial extra-axial tumors (von Spreckelsen et al., 2022). This interface is typically characterized by a meningioma capsule. Nakasu et al. (2005) proposed that this capsule comprises proliferating connective tissue, manifesting as hyperplastic arachnoid trabeculae in the subarachnoid or supra-arachnoid spaces. On magnetic resonance imaging, a well-defined, thick capsule typically indicates benign meningiomas, whereas disrupted capsular integrity often suggests aggressive variants. Multiple studies demonstrate that arachnoid layer disruption, as assessed by MRI, correlates with higher histological grade and increased recurrence risk (Spille et al., 2019). Histopathologically, the capsule covering the brain-facing surface of meningiomas exhibits rich vascularity with elevated vascular endothelial growth factor (VEGF) expression (Uchida et al., 2017). Tumor invasion and subsequent capsular disruption compromise local vasculature, potentially contributing to the development of the “Peritumoral Hyperintense Enhancement Sign.”

The tumor capsule demonstrates dynamic roles during disease progression. During early stages, capsule formation may restrict tumor growth; however, in advanced disease, certain capsular regions may facilitate tumor progression after neoplastic transformation (Rahmanzade, 2020). In our meningioma cohort, the “Peritumoral Hyperintense Enhancement Sign” consistently demonstrates a well-defined border at the tumor interface with a gradual signal attenuation extending toward brain parenchyma. This radiological appearance likely represents either capsular destruction by tumor cells or localized capsular co-option, facilitating brain infiltration.

Per EANO (European Association of Neuro-Oncology) guidelines, surgical resection is recommended for larger meningiomas, while SRS (stereotactic radiosurgery) is preferred for smaller lesions or patients with surgical contraindications (Goldbrunner et al., 2021). The primary surgical objective is maximal safe resection to minimize morbidity and preserve neurological function. The extent of resection depends on tumor location, size, consistency, and involvement of critical neurovascular structures (Vogelbaum et al., 2010). In radiotherapy planning, the CTV (clinical target volume) for IMRT (intensity-modulated radiation therapy) and poRT (postoperative radiotherapy) expands from the GTV (gross tumor volume) to include adjacent involved structures (e.g., thickened meninges, bone; expansion margins: 0 mm for grade I, 5 mm for grade II, 10 mm for grade III). For SRS, the CTV equals the GTV without additional expansion (Martz et al., 2023). The “Peritumoral Hyperintense Enhancement Sign” aids in delineating radiotherapy targets and optimizing surgical margins, thereby improving therapeutic outcomes and postoperative quality of life.

WHO grading and extent of resection are critical predictors of recurrence, both closely tied to brain invasion (Marastoni and Barresi, 2024). Grade II meningiomas with brain invasion exhibit higher progression rates and worse prognoses (Park et al., 2019). Thus, precise identification of the “Peritumoral Hyperintense Enhancement Sign” informs surgical planning, reduces recurrence risk, and enhances patient outcomes.

### 4.2 Brain metastases

Brain metastases, common in advanced systemic malignancies, arise via hematogenous spread and are associated with high mortality (Achrol et al., 2019). Invasion patterns include well-demarcated growth, diffuse infiltration, and vascular proliferation (tumor cell protrusion into perivascular cortical spaces) (Du et al., 2024). Accurate imaging assessment of invasion is vital for treatment selection and prognostication.

CE-MRI (Contrast-enhanced MRI) with gadolinium-based agents is the gold standard for detecting brain metastases, offering superior soft-tissue resolution to differentiate tumor from edema without radiation exposure (Soliman et al., 2018). For solitary metastases, en bloc resection reduces postoperative edema and preserves adjacent brain tissue. Surgical planning relies on preoperative CE-T1WI (CE-T1-weighted imaging) to delineate tumor margins (Soliman et al., 2018). The “Peritumoral Hyperintense Enhancement Sign” guides complete tumor excision while minimizing damage to healthy tissue, thereby reducing surgical morbidity.

Radiotherapy remains the standard treatment for multiple metastases. SRS is recommended for 1–4 lesions, as supported by international clinical trials (Ferguson et al., 2017), WBRT (while whole-brain radiotherapy) is reserved for cases with over 4 metastases. Precise GTV delineation is critical for SRS efficacy. A pivotal study by the ISRS (International Stereotactic Radiosurgery Society) randomized patients with resected brain metastases to postoperative SRS versus observation. Among 64 patients receiving SRS, 12 experienced local failure, with 25% of failures occurring at resection margins in cases with preoperative dural invasion, suggesting expanded CTV margins may improve local control (Mahajan et al., 2017). The “Peritumoral Hyperintense Enhancement Sign,” by demarcating invasive tumor boundaries, refines target volume definition, enhancing SRS precision and patient survival.

## 5 Conclusion

The “Peritumoral Hyperintense Enhancement Sign” represents a valuable imaging biomarker for brain invasion in both meningiomas and metastatic tumors. Brain invasion in the area of abnormal signals is proved by pathology. This sign facilitates more precise treatment planning and may contribute to improved prognostic assessment and reduced recurrence risk.

### 5.1 Limitation

Although the signs presented in this paper were confirmed pathologically, there are deficiencies in the analysis of histological markers associated with brain invasion at the tumor-brain interface, which require follow-up studies for refinement. In addition, the number of cases in this paper is relatively small, which may lead to a certain lack of representativeness in this study.

## Data Availability

The original contributions presented in the study are included in the article/supplementary material, further inquiries can be directed to the corresponding authors.
